# Plasma ACE and ACE2 Levels Are Altered in Patients with COVID-19

**DOI:** 10.3390/v18040465

**Published:** 2026-04-14

**Authors:** Murat Oz, Wassim Chehadeh, Omamah Alfarisi, Farhan S. Cyprian

**Affiliations:** 1 Department of Pharmacology and Therapeutics, College of Pharmacy, Kuwait University, Safat 13110, Kuwait; 2 Department of Microbiology, Faculty of Medicine, Kuwait University, Safat 13110, Kuwait; 3 College of Medicine, QU Health, Qatar University, Doha 2713, Qatar

**Keywords:** ACE, ACE2, COVID-19, renin–angiotensin system

## Abstract

**Objective:** The COVID-19 pandemic has strained healthcare systems and has been associated with substantial morbidity and mortality. Severe acute respiratory syndrome Coronavirus 2 (SARS-CoV-2) enters host cells by binding to angiotensin-converting enzyme 2 (ACE2), implicating dysregulation of the renin–angiotensin system (RAS) in COVID-19 pathophysiology. Measurement of circulating RAS components, including ACE and ACE2, may therefore provide an insight into disease severity and underlying mechanisms. **Subjects and Methods:** In this retrospective cohort study, 224 adults with PCR-confirmed COVID-19 were stratified by World Health Organization disease-severity criteria into asymptomatic, mild, mild-pneumonia, severe, and critical groups. Plasma ACE and ACE2 concentrations were quantified by ELISA. Demographic, clinical, and laboratory data were extracted from electronic medical records. **Results and Conclusions:** Increasing disease severity was associated with higher mortality, elevated body mass index, and higher viral load estimates. Severe and critical illness was characterized by leukocytosis with neutrophilia, marked lymphopenia, anemia, elevated inflammatory and coagulation markers, renal dysfunction, and hypoalbuminemia. Plasma ACE2 levels declined progressively with increasing severity and were significantly lower in patients with mild-pneumonia, severe, or critical illness compared with asymptomatic or mild cases, showing a strong inverse correlation with severity. In contrast, plasma ACE levels increased significantly with disease severity. The resulting increase in the ACE/ACE2 ratio indicates a progressive shift toward the pro-inflammatory arm of the RAS, providing mechanistic insight into the COVID-19 pathophysiology.

## 1. Introduction

The Coronavirus disease-2019 (COVID-19) emerged as a global pandemic affecting millions of individuals and raised great concern throughout the world. The severe acute respiratory syndrome Coronavirus-2 (SARS-CoV-2) was recognized as the causative agent for COVID-19. Importantly, angiotensin-converting enzyme 2 (ACE2) was found to be the entry receptor and a primary target for the entry of SARS-CoV-2 into host cells [1]. The spike protein of SARS-CoV-2 binds to ACE2 and downregulates the expression of ACE2 on the cell surface by promoting the endocytosis of virus-bound ACE2, decreasing the transcription of ACE2 in the infected host cell, and promoting the cleavage of the ACE2 ectodomain by transmembrane serine protease 2 and ADAM17 which causes further downregulation of ACE2 on the host cell surface [1,2].

Several earlier studies about the pathophysiology and epidemiology of the SARS-CoV-2 pandemic suggest that the renin–angiotensin system (RAS) plays a key role in the COVID-19 disease [3,4]. On the classical arm of the RAS pathway, renin mediates the conversion of angiotensinogen to angiotensin I (Ang I). The angiotensin-converting enzyme (ACE), primarily localized in the lungs and to a lesser degree in the kidney, mediates the conversion of Ang I to angiotensin II (Ang-II). On the counterbalance RAS pathway, ACE2 converts Ang-I and Ang-II to Ang-(1–9) and Ang-(1–7), respectively. The latter acts via the Mas receptor to downregulate aldosterone and exert other protective effects on the heart and blood vessels [1]. Downregulation of pulmonary ACE2 expression by SARS-CoV-2 infection and subsequent enhancement of plasma Ang-II levels have been proposed to play a central role in the development of lung and cardiovascular pathologies and promote oxidative stress, inflammation, fibrosis, and increased vascular tone in COVID-19 patients [3]. Dysregulation of RAS by decreased tissue ACE2 levels and proposed overactivation of the classical ACE pathway have prompted several studies investigating the role of the RAS as a disease modifier and whether pharmacological manipulation of the RAS might improve outcomes in patients with COVID-19 [3,4]. Thus, concentrations of soluble ACE2 and ACE in the circulation of COVID-19 patients have been the subject of some recent studies [2]. However, the results of these investigations have not been conclusive. In this study, we investigated the plasma levels of ACE2 and ACE in COVID-19 patients stratified into groups defined by their disease severity: asymptomatic, mild, mild pneumonia, severe, and critical.

## 2. Subjects and Methods

### 2.1. Study Design and Data Collection

This retrospective cohort study includes 224 patients diagnosed with COVID-19 at Hamad Medical Corporation in Qatar. The sample size was determined by the availability of biological samples and corresponding clinical data during the study period, with an intended target of approximately 40–50 patients per disease severity group to allow for meaningful comparative analyses. Patients were 86.2% males, with ages ranging from 35 to 69 years, and presented with various co-morbidities, including hypertension, diabetes, asthma, cardiovascular disease, liver disease, and kidney disease. The mean age increased with disease severity (see Table 1). The total cohort was divided into five groups according to the WHO guidelines (https://apps.who.int/iris/bitstream/handle/10665/349321/WHO-2019-nCoV-clinical-2021.2-eng.pdf, accessed on 20 December 2025). Briefly, the groups were as follows: the first group included asymptomatic patients diagnosed with COVID-19 (*n* = 38), the second group included patients with mild symptoms (*n* = 41), the third group included patients with mild pneumonia (*n* = 41), the fourth group included those with severe pneumonia requiring oxygen support (*n* = 48), and the fifth group included critically ill ICU cases on ventilatory support (*n* = 56). SARS-CoV-2 infection was diagnosed by the TaqPath COVID-19 Combo Kit (Thermo Fisher Scientific, Waltham, MA, USA) or Cobas SARS-CoV-2 Test (Roche Diagnostics, Rotkreuz, Switzerland). Upper respiratory tract specimens used in diagnostic kits were obtained through throat and nasopharyngeal swabs. Blood samples were collected in EDTA-containing tubes and subsequently centrifuged at 1500 *g* for 10 min at room temperature (22–24 °C); aliquoted plasma samples were stored at −80 °C until analysis. The study was performed in accordance with the guidelines of the Declaration of Helsinki, and ethical approvals were obtained from the Institutional Review Board (IRB) of Hamad Medical Corporation (Doha, Qatar) (MRC-05-084).

Plasma samples for ACE and ACE2 measurements were collected concurrently with routine clinical laboratory tests (i.e., at the same time as the laboratory samples) and data is presented in Table 2. For asymptomatic and mild cases, this occurred at the time of PCR confirmation (typically within 24 h of diagnostic swab). For hospitalized patients (mild-pneumonia, severe, and critical groups), samples were obtained within 24 h of admission or ICU transfer. This standardized acute-phase sampling minimizes variability related to disease duration at the time of biomarker assessment.

COVID-19 patients with clinically mild pneumonia to severe disease symptoms were hospitalized for in-patient management. Patients in the clinically severe group were admitted to the ICU, and blood samples were collected at the time of ICU admission. Standard treatment for hospitalized patients comprised supportive care, antiviral therapy, and individual regimens according to the severity of the disease and the presence of comorbidities. Clinical and laboratory data included body mass index (BMI), diastolic blood pressure, and viral load, among others. Routine blood tests comprised complete blood cell counts, glucose, electrolytes, total protein, albumin, C-reactive protein, IL-6, procalcitonin, D-dimers, urea, ferritin, and liver enzymes, and were retrieved from the electronic healthcare system of the Hamad Medical Corporation. Renal function tests were conducted for all hospitalized COVID-19 cases.

### 2.2. Measurements of Enzyme Concentrations

Plasma concentrations of ACE and ACE2 were determined using commercially available enzyme-linked immunosorbent assay (ELISA) kits (Cat # MBS2701265 for ACE and MBS264649 for ACE2, Mybiosource, San Diego, CA, USA). The measurement was performed according to the instructions in the manual. We used a 10-fold dilution of the samples prior to measurement. All samples and standards were assayed in triplicate. The absorbance of the samples was measured on a microplate reader (BioTeck Instruments, Inc., Winooski, VT, USA). Concentrations were calculated from a standard curve. For samples with ACE2 levels below the lower limit of detection, a value of 1 pg/mL was imputed for the purpose of calculating the ACE/ACE2 ratio.

### 2.3. Statistical Analysis

The primary endpoint was the plasma concentration of the enzymes ACE and ACE2. Variables were explored first for normal distribution and equality of variances using the Shapiro–Wilk normality test and Brown–Forsythe test, respectively. The results indicated that nonparametric tests should be used for statistical analysis. Consequently, continuous variables were compared using the Mann–Whitney U test or Kruskal–Wallis test, as appropriate. When the Kruskal–Wallis test was used, *p*-values for pairwise comparisons were adjusted using Dunn’s post hoc test with Bonferroni adjustment. Kendall’s tau-b correlation test was run to determine the relationship between an ordinal variable (i.e., the severity of the disease) and a continuous variable (i.e., ACE or ACE2 levels). Correlation coefficients are presented together with their 95% confidence intervals. A 2-sided *p*-value < 0.05 was considered statistically significant. Data are represented as median (interquartile range) for non-normally distributed variables or mean +/− standard deviation for demographic data. All statistical analyses were performed using the IBM SPSS Statistics 25 statistical package (IBM Corporation, Armonk, NY, USA).

## 3. Results

A total of 224 patients who met the criteria were included in our retrospective study, of which 86.2% were male and 13.8% were female. Demographic characteristics of the patients are presented in Table 1. An increasing disease severity was associated with a higher mean age and COVID-19 Ct value, as well as a greater proportion of male patients, while mortality was markedly higher in the severe and critical patient groups, as depicted in Table 1. The study population was 86.2% male, consistent with the higher hospitalization rates among men during the pandemic, as well as demographics in Qatar (and cultural limitations of obtaining patient consent).

The prevalence of key comorbidities (hypertension, diabetes mellitus, cardiovascular disease, chronic kidney disease, and chronic liver disease) stratified by disease severity is presented in Supplementary Table S1. Comorbidities were more prevalent in the mild-pneumonia, severe, and critical groups.

The laboratory parameters summarized in Table 2 further demonstrate that severe and critical illness are characterized by leukocytosis, neutrophilia, profound lymphopenia, elevated inflammatory markers (C-reactive protein, IL-6, procalcitonin, ferritin), coagulation abnormalities (elevated D-dimers), renal dysfunction (elevated urea), and hypoalbuminemia. Notably, an increased severity of COVID-19 was associated with elevated serum glucose and triglyceride levels (Table 2) observed in patients with diabetes and hypertension.

**Figure 1:**
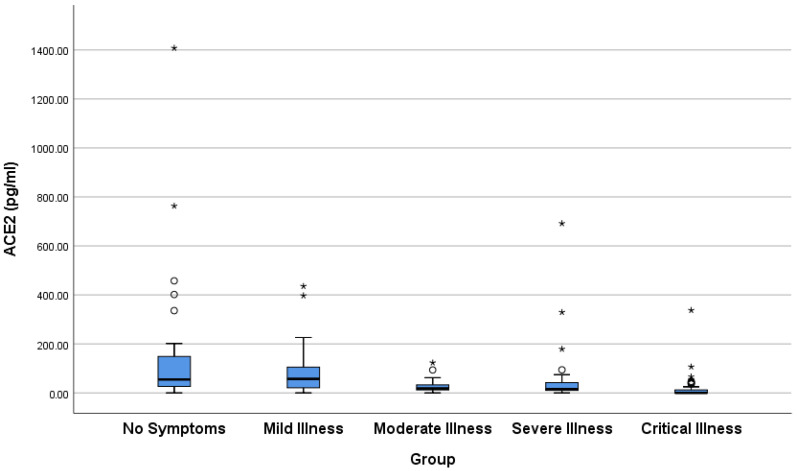
Box-and-whisker plot showing the distribution of ACE2 levels in different groups of patients with COVID-19. The lower and upper boundaries of the box indicate the 25th and 75th percentiles, respectively. The black line within the box marks the median. Whiskers above and below the box indicate 1.5 IQR (interquartile range). Small circles and stars outside the whiskers’ boundaries indicate outliers.

Patients with mild-pneumonia, severe, or critical symptoms had statistically significant lower levels of ACE2 than those with no symptoms (Table 3, Figure 1) or mild symptoms (*p* range: <0.001–0.01). In addition, patients with critical COVID-19 had statistically significant lower levels of ACE2 than those with mild pneumonia (*p* = 0.021) or severe COVID-19 (*p* = 0.006). Overall, there was a significant inverse correlation between ACE2 levels and the severity of the disease (τb = −0.438, *p* < 0.001).

**Figure 2:**
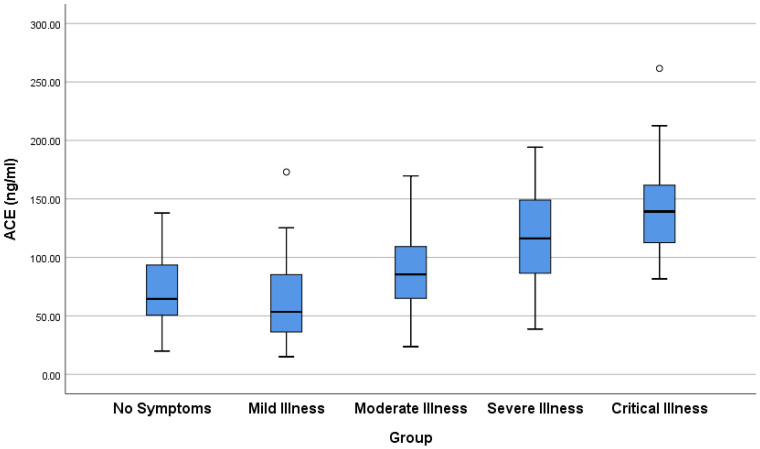
Box-and-whisker plot showing the distribution of ACE levels in different groups of patients with COVID-19.

Patients with severe or critical COVID-19 had statistically significant higher levels of ACE than those with no or mild symptoms (*p* < 0.0001; Table 4, Figure 2). The ACE levels in patients with mild COVID-19 symptoms were not statistically different from those in patients with no or mild-pneumonia (moderate) symptoms. There was no statistical difference in ACE levels between patients with severe COVID-19 and those with mild pneumonia (*p* = 0.052) or critical COVID-19 (*p* = 0.063). However, there was a strong positive correlation between ACE level and severity of the disease, which was statistically significant (τb = 0.480, *p* < 0.0001).

Patients with mild-pneumonia (moderate), severe, or critical symptoms had statistically significant higher ACE/ACE2 ratios than those with no or mild symptoms (*p* ≤ 0.001). As summarized in Table 4, the ACE/ACE2 levels in patients with moderate COVID-19 symptoms were not statistically different from those in patients with severe symptoms (*p* = 1.00), but the ratio in patients with moderate symptoms was significantly lower than that in patients with critical symptoms (*p* < 0.001). Patients with critical symptoms had statistically significant higher ACE/ACE2 ratios than those with severe illness (*p* = 0.002). There was a strong positive correlation between the ACE/ACE2 ratio and the severity of the disease, which was statistically significant (τb = 0.505, *p* < 0.001).

## 4. Discussion

**Figure 3:**
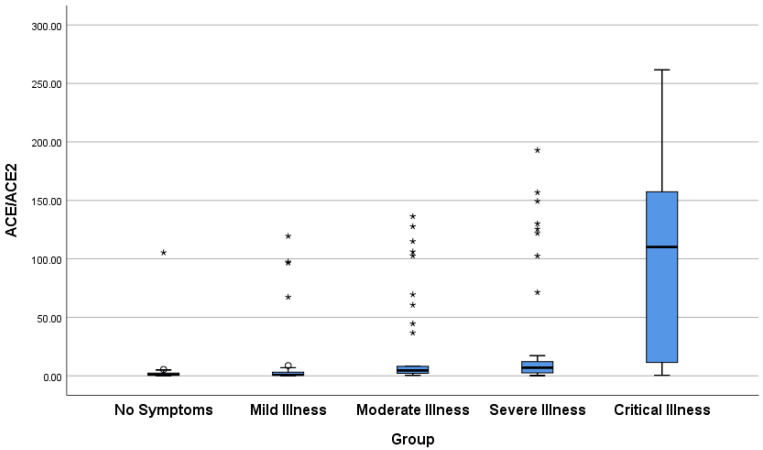
Box-and-whisker plot showing the distribution of the ACE/ACE2 levels in different groups of patients with COVID-19. Undetectable ACE2 levels were imputed with a value of 1 to allow for the ratio calculation.

Following the COVID-19 pandemic, several in vivo and in vitro studies have analyzed the interaction between ACE2 and SARS-CoV-2 [1,2]. Consequently, several clinical research teams have proposed that in COVID-19 patients, SARS-CoV-2 triggers downregulation of ACE2, leading to alterations in the balance between Ang II and Ang (1–7); contributing to the increased arterial resistance, oxidative stress, and release of proinflammatory cytokines; and accelerating the deterioration of the cardiovascular and immune systems. ACE2 acts in an opposing manner to its homolog, ACE, by inactivating the vasoconstrictor peptide Ang II and generating the vasodilator peptide, Ang-(1–7) [2,4,5]. In this study, we report evidence that plasma concentrations of ACE2 and ACE are significantly altered during COVID-19 in a severity-dependent manner. Specifically, plasma ACE2 concentration declined markedly with COVID-19 severity, as illustrated in Table 3 and Figure 1. Patients with mild-pneumonia, severe, or critical symptoms had significantly lower ACE2 levels than asymptomatic or mild cases. In general, ACE2 levels showed a strong inverse correlation with illness severity. On the other hand, plasma ACE concentrations showed a marked positive relationship with disease severity (Table 4, Figure 2). Thus, severe and critical patients had significantly higher plasma ACE than asymptomatic or mild cases. Strikingly, the ACE/ACE2 ratio, a putative index of RAS imbalance, increased dramatically with disease severity (Table 5, Figure 3). In conclusion, our findings suggest that severe COVID-19 is marked by the systemic loss of ACE2 and a reciprocal rise in ACE, culminating in a profoundly altered ACE/ACE2 balance.

In an earlier study, a significant decrease (70%) of serum ACE2 levels was reported in male COVID-19 patients, and ACE2 levels were inversely correlated with disease severity [6]. Another study reported a transient increase in serum ACE2 levels but no change in ACE concentrations in COVID-19 patients [7]. Our findings of decreased soluble ACE2 in severe COVID-19 align with several previous reports [8,9,10,11,12,13]. The observed decline may reflect several mechanisms such as increased shedding of the membrane-bound enzyme into tissues, internalization of the ACE2–spike complex, and/or reduced expression due to virus-induced cellular damage. The significant inverse correlation with clinical severity supports the hypothesis that loss of ACE2-mediated protective signaling (via Ang 1–7) is a hallmark of progressive disease. Conversely, some studies have reported no change [14,15,16] or marked elevations in serum ACE2 levels in COVID-19 patients [5,17]. These discrepancies may be attributed to the differences in study populations, phase of the patient’s illness (acute versus convalescent phase), assay methodology, or specific ACE2 isoforms measured (i.e., full length versus truncated).

Regarding ACE, we observed a clear positive correlation between its plasma concentration and disease severity. Others have reported a decrease in ACE activity but not in plasma concentration [18]. Similarly, hospitalized patients with COVID-19 were found to have lower serum ACE activity than controls [19]. Notably, ACE activity was reported to be reduced in patients with COVID-19 Acute Respiratory Disease Syndrome (ARDS) compared to non-COVID-19 ARDS, especially in patients who did not survive [20] and in patients with severe versus non-severe COVID-19 [21]. In our study, we found a statistically significant increase in serum ACE levels across all COVID-19 cohorts. This finding is consistent with the report by Tepasse et al. (2022) of high serum ACE activity correlating with severity [22]. Elevated circulating ACE could lead to increased generation of the pro-inflammatory, pro-fibrotic, and vasoconstrictive peptide ANG II, thereby exacerbating lung injury and endothelial dysfunction, leading to local and systemic inflammation. Our results contrast with studies reporting decreased ACE activity [18,19,20,21], highlighting that enzymatic activity and immunoreactive protein concentration may not always correlate perfectly due to the presence of endogenous inhibitors or conformational changes. In addition, elevated serum ACE levels in patients with post-acute COVID-19 syndrome were reported [23]. While a significant increase in serum ACE levels in convalescent plasma donors with SARS-CoV-2 infection was reported [24], other studies found no change in serum ACE concentrations in COVID-19 patients [7,15,16].

**Figure 4:**
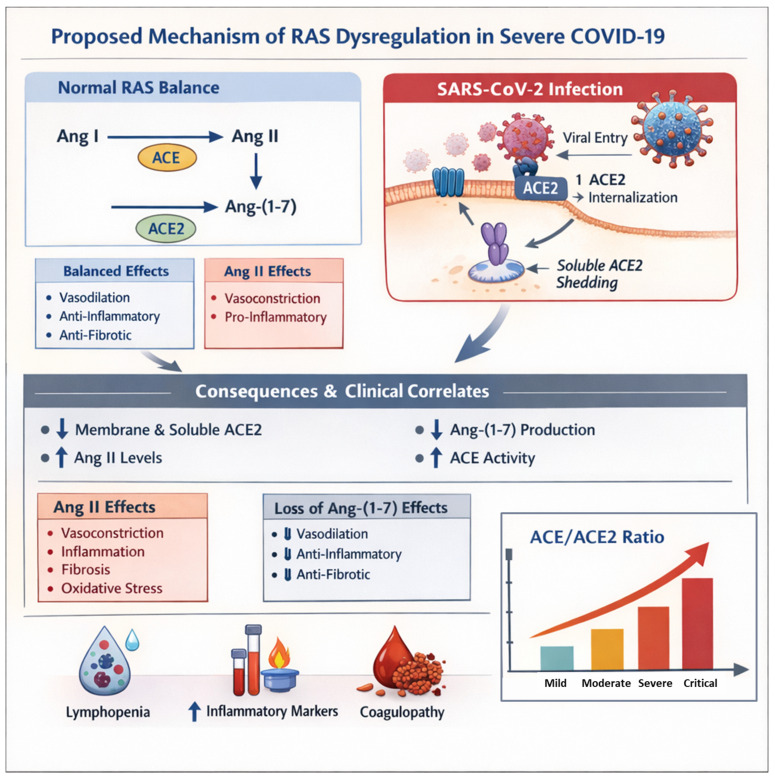
Proposed mechanism of renin–angiotensin system (RAS) dysregulation in severe COVID-19: Under physiological conditions, a balanced RAS is maintained through ACE-mediated conversion of angiotensin I (Ang I) to angiotensin II (Ang II) and ACE2-mediated generation of angiotensin-(1–7) [Ang-(1–7)], resulting in vasodilatory, anti-inflammatory, and anti-fibrotic effects. During SARS-CoV-2 infection, viral binding to membrane-bound ACE2 leads to ACE2 internalization and shedding, reducing both membrane and soluble ACE2 availability. This disrupts Ang-(1–7) production and favors increased ACE activity and Ang II accumulation. The resulting RAS imbalance promotes vasoconstriction, inflammation, fibrosis, and oxidative stress, while protective Ang-(1–7)-mediated effects are diminished. These molecular alterations are associated with key clinical correlations of severe COVID-19, including lymphopenia, elevated inflammatory markers, coagulopathy, and a progressive increase in the ACE/ACE2 ratio with disease severity.

The most robust finding in our cohort is the dramatic, severity-dependent increase in the ACE/ACE2 ratio. The strong ordinal correlations across the five severity strata (Kendall’s τb) support a progressive, severity-dependent shift toward the pro-inflammatory ACE/Ang II axis, consistent with SARS-CoV-2-induced ACE2 internalization and shedding. This ratio integrates the opposing arms of the RAS and may provide a more sensitive biomarker of RAS dysregulation than either enzyme alone. As shown in Table 5, the median ACE/ACE2 ratio was 1.16 in asymptomatic cases but markedly increased to 110.17 in critically ill patients. This shift strongly implies a profound imbalance favoring the pro-inflammatory, pro-injurious ACE/Ang II/AT1R axis over the protective ACE2/Ang (1–7)/MasR axis. Such an imbalance is consistent with reported elevations in Ang II in severe COVID-19 [12,25,26,27,28] and provides a plausible mechanistic link to the cytokine storm, coagulopathy and multi-organ failure observed in advanced stages of the disease. This profound shift in the RAS balance is illustrated in our proposed mechanistic model (Figure 4). Similarly, serum Ang-II levels were higher in severe than non-severe COVID-19 patients [21]. However, a significant decrease [15,29] or no alterations [14,16] in serum Ang-II levels of COVID-19 patients were also reported.

Critically ill patients showed higher potassium levels in the context of markedly elevated urea and creatinine (Table 2), consistent with renal dysfunction rather than a direct RAS-mediated effect on potassium homeostasis.

Leukocytosis, neutrophilia, and elevated procalcitonin in severe and critical groups may suggest concomitant bacterial infection. While such infections can further activate inflammatory pathways that modulate RAS components, the observed ACE/ACE2 alterations remained strongly correlated with WHO clinical severity even after accounting for these markers.

The kallikrein–kinin system (KKS) acts as a physiological counterbalance to the RAAS, promoting vasodilation, reducing oxidative stress, and protecting against organ damage in the heart and kidneys. SARS-CoV-2-induced dysregulation of the RAAS may also impair KKS activity, further exacerbating inflammation and tissue injury; this interplay warrants further investigation. In addition to RAS, ACE2 and ACE play roles in the regulation of KKS. While ACE converts bradykinin to inactive fragments, the ACE2 degrades des-Arg9-bradykinin to pharmacologically inactive peptides [30]. Notably, in line with the increased plasma ACE levels observed in our study, decreased bradykinin levels in COVID-19 patients have been reported in several earlier studies [13,31,32].

Tissue ACE2 is anchored to the cell membranes in the lungs (type II pneumocytes), small intestines, kidneys, heart, and blood vessels. Since tissue ACE2 acts as the binding site for the SARS-CoV-2 spike protein, allowing the virus to enter the cell, high tissue expression generally correlates with higher susceptibility to infection in target organs. Subsequent to SARS-CoV-2 binding, tissue ACE2 levels have been shown to be downregulated, disrupting the RAS balance and leading to increased inflammation, vasoconstriction, and tissue damage [1,33].

A soluble form of ACE2, found in the plasma, is produced when transmembrane tissue ACE2 is cleaved by enzymes like ADAM17 (shedding) or TMPRSS2. It has been proposed that circulating ACE2 functions as a “decoy receptor” by binding to the virus in the circulation and preventing it from binding to tissue-bound ACE2. Thus, high plasma ACE2 levels have been proposed to be associated with neutralization of the virus in some studies [34]. However, other clinical studies indicate that high levels of serum ACE2 are paradoxically associated with higher mortality or disease severity, likely because it indicates widespread tissue damage and massive shedding [33,34,35].

Although no direct correlation between plasma ACE2 activity and ACE2 levels in heart or lung tissue has been established [34], elevated plasma ACE2 levels have been consistently associated with systemic inflammation and the severity of conditions like cardiovascular disease or acute COVID-19 infection, rather than reflecting local tissue expression levels [34,35].

It appears that in cardiovascular pathologies where RAS dysregulation occurs, the interaction between ACE and ACE2 is reciprocal; when one of them is upregulated, the other one is downregulated [3]. Based on the findings in this study, we propose an ACE-Index (ACE/ACE2 ratio) as a compact prognostic metric that, once validated and combined with inflammatory/coagulation markers (e.g., CRP, IL-6, C5a) and patient demographics, may improve early identification of patients at risk of clinical deterioration. Integration of the ACE/ACE2 ratio with established clinical and laboratory predictors such as lymphocyte count, inflammatory markers, and comorbidities could enhance clinical risk stratification and aid in early identification of patients at higher risk of severe COVID-19. The proposed dysregulation linking these molecular changes to clinical outcomes is summarized in Figure 4.

Although comorbidities were more frequent in the mild-pneumonia, severe, and critical groups (Supplementary Table S1), hypertension and diabetes are typically associated with elevated (not reduced) circulating ACE2 levels in previous studies. Similarly, ACEi/ARB therapy—commonly prescribed in these patients—has not been shown to meaningfully alter plasma soluble ACE2 concentrations in COVID-19 cohorts. The observed severity-dependent decrease in ACE2 and reciprocal increase in the ACE/ACE2 ratio are therefore unlikely to be explained by these factors alone.

In addition, gender can be a confounding factor since serum ACE2 concentrations have been reported to be elevated in men. Plasma ACE2 levels also change with age, generally following an inverted U-shaped curvilinear association: higher in infancy, lower during childhood and early adulthood, and then potentially increasing again or showing a different trend later in life [17,36], which limits the contribution of age as a confounding factor in our findings. Furthermore, the number of comorbid pathologies such as diabetes mellitus, hypertension, and heart diseases was higher in mild-pneumonia, severe, and critically ill groups compared to asymptomatic and mild groups. However, these pathologic conditions are usually associated with increased [5,35,37] but not decreased plasma ACE2 levels, which was demonstrated in our study. In addition, hypertensive drugs used in different patient groups have been shown not to alter ACE2 levels in several earlier studies [4,16,35].

This study has several limitations. Its retrospective design precludes establishing causality between RAS changes and disease outcomes, and the cross-sectional measurements prevent tracking temporal shifts in ACE and ACE2 levels. While our cross-sectional design precludes intra-individual trajectory modeling, the clear severity-dependent gradients observed provide mechanistic support for RAS imbalance in progressive disease. Imputing undetectable ACE2 values introduces some uncertainty in ratio calculations (this is more frequent in severe/critical groups); however, sensitivity analyses confirmed the direction and statistical significance of all findings. Due to the retrospective design, detailed patient-level medication records (including ACEi/ARB use at the exact time of sampling) were not uniformly available; we were therefore unable to perform quantitative adjustment for these drugs. The cohort was predominantly male (86.2%) and hospital-based. The ACE2 gene is located on the X chromosome, and sex differences in ACE2 expression and regulation have been reported; however, the inverse relationship between ACE2 and disease severity persisted across the cohort. Hospital-based recruitment may enrich for more severe presentations, limiting generalizability to community-managed or female-predominant populations. Future studies in more balanced cohorts are warranted.

The strength of this study lies in the clear stratification of patients according to standardized WHO criteria and simultaneous measurement of ACE and ACE2, allowing the calculation of their ratio. The ACE/ACE2 ratio showed a strong positive correlation with disease severity (Kendall’s τb = 0.505, *p* < 0.001) and integrates the opposing arms of the RAS. It may serve as a promising index of RAS imbalance that may serve as an adjunct prognostic marker pending validation. Future prospective studies are needed to validate the ACE/ACE2 ratio as a tool for early risk stratification and to investigate whether therapeutic strategies aimed at restoring RAS balance could improve the outcomes in patients with severe COVID-19.

## 5. Conclusions

In conclusion, our findings indicated that plasma ACE2 levels showed a strong inverse correlation with illness severity (Figure 1). On the other hand, plasma ACE levels showed a marked positive relationship with disease severity. These reciprocal changes define a systemic RAS shift consistent with loss of the protective ACE2 axis and activation of ACE-driven pathways implicated in inflammation, endothelial dysfunction, and organ injury. The resultant increase in the ACE/ACE2 ratio provides a quantitative measure of imbalance correlation with disease severity. Our findings indicate that severe COVID-19 is characterized by the systemic loss of ACE2 accompanied by a reciprocal increase in ACE, resulting in marked dysregulation of the renin–angiotensin system. A schematic summary of this pathogenic mechanism, linking SARS-CoV-2-induced ACE2 loss to clinical severity, is shown in Figure 4. Prospective, multi-center studies with serial measurements and external validation cohorts are needed to establish the ACE/ACE2 ratio as a reliable prognostic biomarker and to determine whether therapeutic targeting of RAS imbalance can improve outcomes in severe COVID-19. Future studies should validate the prognostic utility of the ACE/ACE2 ratio in independent, prospective cohorts and test multivariate models incorporating the ratio together with established predictors (lymphocyte count, CRP, IL-6, D-dimer, and comorbidities) for risk stratification or outcome prediction (mortality, ICU admission).

## Figures and Tables

**Table 1:** Patient demographics diagnosed with Sars-CoV-2 viral infection. Baseline demographic and anthropometric characteristics of the study population stratified by COVID-19 disease severity.

Characteristic	N	Asymptomatic ^1^	Mild Symptomatic ^1^	Mild Pneumonia ^1^	Severe ^1^	Critical ^1^	*p*-Value ^2^
**Living status**	224						**<0.001**
**Alive**		38 (21%)	41 (23%)	40 (22%)	42 (23%)	19 (11%)	
**Dead**		0 (0%)	0 (0%)	1 (2.3%)	6 (14%)	37 (84%)	
**Gender**	224						**0.043**
**Female**		3 (8.1%)	9 (24%)	12 (32%)	4 (11%)	9 (24%)	
**Male**		35 (19%)	32 (17%)	29 (16%)	44 (24%)	47 (25%)	
**Height [m]**	150	164 (158–169)	164 (161–171)	166 (161–174)	168 (165–172)	165 (158–170)	0.3
**Weight [kg]**	152	72 (59–83)	76 (64–86)	86 (73–95)	80 (70–90)	77 (69–90)	**0.039**
**Body Mass Index (BMI) [kg/m^2^]**	141	24.4 (22.3–27.1)	27.5 (24.0–31.8)	29.2 (27.3–34.1)	27.6 (25.3–30.1)	27.3 (25.0–30.1)	**0.010**
**COVID-19 Average CT**	182	20.8 (18.3–26.3)	22.2 (18.2–28.6)	26.6 (18.8–32.0)	29.0 (24.1–34.0)	27.4 (22.0–30.2)	**<0.001**

^1^ Median (Q1–Q3); n (%). ^2^ Kruskal–Wallis rank sum test; Fisher’s exact test. Data are presented as median (interquartile range, Q1–Q3) for continuous variables and number (percentage) for categorical variables. Disease severity categories include asymptomatic, mild symptomatic, mild pneumonia, severe, and critical illness (WHO clinical criteria). N indicates the number of participants with available data for each characteristic. *p*-values represent overall comparisons across severity groups using the Kruskal–Wallis rank sum test for continuous variables and Fisher’s exact test for categorical variables. Living status refers to the patient outcome (alive or dead) at the end of follow-up.

**Table 2:** Patients’ clinical parameters following SARS-CoV-2 viral infection. Clinical hematological, inflammatory, coagulation and biochemical laboratory parameters of patients stratified by COVID-19 disease severity.

Characteristic	N	Asymptomatic ^1^	Mild Symptomatic ^1^	Mild Pneumonia ^1^	Severe ^1^	Critical ^1^	*p*-Value ^2^
**Living status**	224						**<0.001**
**White blood cell count (WBC) [×10^3^/μL]**	221	6.5 (5.4–7.5)	5.9 (4.6–7.4)	6.4 (4.4–10.6)	9.1 (7.4–12.2)	13.5 (8.9–21.4)	**<0.001**
**Red blood cell count (RBC) [×10^6^/μL]**	221	5.20 (4.85–5.40)	5.10 (4.70–5.50)	4.90 (4.50–5.60)	4.05 (3.45–4.60)	3.10 (2.70–3.75)	**<0.001**
**Hemoglobin (Hgb) [g/dL]**	221	14.95 (13.90–15.50)	14.45 (13.25–15.25)	13.40 (12.40–14.60)	12.00 (9.80–13.50)	8.80 (8.00–10.55)	**<0.001**
**Hematocrit (Hct) [%]**	221	44 (42–47)	42 (39–46)	40 (38–44)	36 (30–39)	28 (25–33)	**<0.001**
**Mean corpuscular volume (MCV) [fL]**	221	86 (83–89)	83 (80–86)	84 (76–88)	87 (84–91)	89 (87–92)	**<0.001**
**Mean cell hemoglobin (MCH) [pg]**	221	28.50 (27.50–29.95)	27.95 (26.65–29.30)	27.90 (24.80–29.90)	29.45 (28.10–30.10)	29.00 (27.35–30.00)	**0.006**
**Mean corpuscular hemoglobin concentration (MCHC) [g/dL]**	221	33.25 (32.55–34.05)	33.15 (32.25–34.10)	33.30 (32.00–34.10)	33.30 (32.60–34.25)	32.10 (31.40–33.15)	**<0.001**
**Absolute neutrophil count (ANC) [×10^3^/μL]**	221	3.5 (2.6–4.5)	3.3 (2.1–4.5)	4.1 (2.7–7.3)	7.4 (5.4–10.7)	10.9 (7.7–18.8)	**<0.001**
**Neutrophil [%]**	190	57 (50–61)	54 (46–64)	66 (53–76)	81 (74–90)	89 (77–92)	**<0.001**
**Lymphocyte count [×10^3^/μL]**	221	1.80 (1.45–2.40)	2.00 (1.40–2.33)	1.40 (1.10–1.80)	1.10 (0.50–1.55)	0.90 (0.50–1.45)	**<0.001**
**Lymphocyte [%]**	221	30 (25–36)	34 (28–39)	23 (14–38)	11 (6–16)	6 (4–14)	**<0.001**
**Monocyte count [×10^3^/μL]**	221	0.60 (0.50–0.70)	0.50 (0.35–0.70)	0.60 (0.40–0.70)	0.60 (0.40–0.80)	0.65 (0.40–0.90)	0.4
**Monocyte [%]**	221	9.0 (7.6–12.1)	9.1 (7.6–11.6)	8.0 (6.0–10.4)	6.0 (3.9–9.1)	4.8 (3.1–6.9)	**<0.001**
**Eosinophil count [×10^3^/μL]**	221	0.10 (0.00–0.20)	0.10 (0.00–0.17)	0.00 (0.00–0.10)	0.00 (0.00–0.20)	0.00 (0.00–0.10)	**0.007**
**Eosinophil [%]**	221	1.65 (0.65–3.20)	1.20 (0.25–2.10)	0.40 (0.00–1.10)	0.15 (0.00–2.40)	0.20 (0.00–0.90)	**<0.001**
**Basophil count [×10^3^/μL]**	221	0.030 (0.020–0.050)	0.030 (0.015–0.050)	0.020 (0.010–0.030)	0.030 (0.010–0.050)	0.040 (0.020–0.070)	**0.013**
**Basophil [%]**	221	0.50 (0.35–0.75)	0.55 (0.30–0.85)	0.30 (0.20–0.50)	0.30 (0.15–0.50)	0.25 (0.20–0.45)	**<0.001**
**Platelet [×10^9^/L]**	199	246 (218–287)	265 (201–316)	244 (201–354)	322 (242–376)	308 (194–385)	0.080
**Mean platelet volume (MPV) [fL]**	209	10.15 (9.50–11.10)	10.30 (9.40–11.10)	10.45 (10.05–11.00)	10.20 (9.50–11.00)	11.30 (10.55–12.45)	**<0.001**
**Platelet distribution width (PDW) [fL]**	139	14.1 (11.9–16.5)	12.2 (10.2–13.7)	13.5 (11.2–15.9)	11.7 (10.3–13.4)	14.9 (12.5–16.6)	**<0.001**
**Red blood cell distribution width (RDW-CV) [%]**	220	12.30 (11.95–12.95)	12.70 (12.10–14.00)	13.60 (12.70–14.90)	14.15 (12.75–15.30)	17.40 (14.80–19.50)	**<0.001**
**Prothrombin time (PT) [s]**	134	NA	11.10 (11.00–11.70)	11.50 (11.10–12.30)	12.75 (11.90–14.30)	13.45 (12.15–15.30)	**<0.001**
**International Normalized Ratio (INR)**	134	NA	1.00 (1.00–1.10)	1.00 (1.00–1.10)	1.10 (1.00–1.20)	1.10 (1.00–1.30)	**0.014**
**D-Dimer [mg/L FEU]**	158	0.30 (0.24–0.36)	0.39 (0.30–0.61)	0.46 (0.35–0.69)	1.47 (0.78–3.22)	4.23 (1.95–5.80)	**<0.001**
**Fibrinogen [g/L]**	108	NA	NA	4.60 (3.90–5.60)	4.20 (3.30–4.80)	3.50 (2.55–4.80)	0.071
**Partial thromboplastin time (APTT) [s]**	134	NA	28 (27–31)	31 (27–33)	29 (26–34)	34 (30–43)	**0.003**
**C-reactive protein (CRP) [mg/L]**	220	3 (2–9)	4 (2–10)	21 (6–64)	29 (10–83)	67 (33–121)	**<0.001**
**Procalcitonin [ng/mL]**	125	0.0	0.1 (0.0–0.2)	0.2 (0.1–0.4)	0.2 (0.1–0.5)	0.6 (0.3–3.1)	**<0.001**
**Ferritin [μg/L]**	155	256	181 (41–327)	518 (140–880)	752 (458–1360)	1057 (582–2214)	**<0.001**
**Interleukin-6 (IL-6) [pg/mL]**	108	NA	14 (3–34)	24 (9–51)	28 (13–76)	87 (36–146)	**<0.001**
**Urea [mmol/L]**	222	4 (3–4)	4 (3–5)	5 (4–7)	7 (6–12)	16 (10–29)	**<0.001**
**Creatinine [μmol/L]**	220	82 (71–90)	73 (60–83)	78 (57–90)	69 (59–87)	97 (57–239)	**0.032**
**Bilirubin [mg/dL]**	204	8 (5–13)	6 (5–9)	7 (5–13)	9 (6–13)	12 (8–22)	**<0.001**
**Total Protein [g/L]**	196	77 (70–79)	73 (72–77)	69 (66–74)	68 (63–74)	64 (57–73)	**<0.001**
**Albumin [g/L]**	213	42 (38–44)	39 (36–41)	33 (30–36)	26 (24–30)	24 (20–27)	**<0.001**
**Alkaline phosphatase (ALP) [U/L]**	206	82 (74–94)	65 (59–82)	89 (64–97)	97 (74–132)	131 (92–281)	**<0.001**
**Alanine aminotransferase (ALT) [U/L]**	203	25 (19–38)	23 (20–43)	33 (20–64)	48 (26–74)	38 (20–76)	**0.019**
**Aspartate aminotransferase (AST) [U/L]**	185	27 (19–35)	22 (18–29)	28 (21–43)	35 (24–58)	46 (29–76)	**<0.001**
**Sodium [mmol/L]**	222	138 (136–139)	138 (135–140)	136 (134–138)	138 (136–140)	139 (135–145)	**0.015**
**Potassium [mmol/L]**	215	4.80 (4.30–5.10)	4.40 (3.90–4.90)	4.10 (3.80–4.30)	4.20 (3.80–4.40)	4.45 (3.90–4.90)	**<0.001**
**Chloride [mmol/L]**	215	100.0 (98.0–102.0)	101.5 (99.0–103.0)	101.0 (99.0–103.0)	102.0 (100.0–106.0)	103.0 (97.5–107.5)	0.051
**Bicarbonate [mmol/L]**	213	26.0 (25.0–28.0)	26.0 (23.0–28.0)	24.0 (21.0–25.0)	25.0 (23.0–27.2)	27.0 (23.0–30.0)	**0.004**
**Calcium [mmol/L]**	210	2.34 (2.30–2.41)	2.33 (2.22–2.41)	2.19 (2.14–2.33)	2.16 (2.09–2.29)	2.12 (2.06–2.22)	**<0.001**
**Adjusted calcium [mmol/L]**	210	2.32 (2.26–2.36)	2.33 (2.27–2.38)	2.35 (2.29–2.45)	2.45 (2.37–2.54)	2.49 (2.36–2.62)	**<0.001**
**Phosphorus [mmol/L]**	113	NA	1.01 (0.93–1.36)	1.15 (1.11–1.20)	1.06 (0.94–1.25)	1.31 (1.04–1.62)	**0.009**
**Magnesium [mmol/L]**	143	0.84 (0.79–0.87)	0.87 (0.83–0.89)	0.89 (0.79–1.15)	0.92 (0.83–0.97)	0.93 (0.87–0.99)	**0.011**
**Lactate dehydrogenase (LDH) [U/L]**	117	218 (218–218)	256 (180–272)	275 (201–343)	411 (325–487)	448 (357–547)	**<0.001**
**High-sensitivity Troponin-T [ng/mL]**	109	6	9 (4–10)	9 (6–10)	13 (8–22)	106 (38–189)	**<0.001**
**Creatine kinase (CK) [U/L]**	123	97	117 (54–325)	75 (65–206)	64 (44–191)	101 (63–295)	0.3
**Glucose**	198	5.0 (4.5–5.9)	5.9 (5.1–6.9)	6.3 (5.3–8.4)	8.1 (6.5–10.0)	8.8 (6.5–10.9)	**<0.001**
**Hemoglobin A1C (HbA1C) [%]**	121	7.40 (5.70–9.30)	6.65 (5.80–8.70)	6.85 (5.50–9.70)	6.80 (6.25–8.60)	5.90 (5.60–7.70)	0.5
**Cholesterol**	94	4.00 (2.76–4.63)	3.75 (3.30–4.40)	4.40 (3.48–5.57)	4.05 (3.40–5.57)	3.60 (2.80–4.70)	0.3
**Low-density lipoprotein (LDL) [mmol/L]**	19	NA	1.99 (1.78–2.20)	2.14 (2.01–4.15)	3.17 (3.05–3.43)	2.03 (1.63–2.52)	0.15
**Triglyceride [mmol/L]**	142	0.80 (0.77–0.92)	1.20 (1.10–1.60)	1.56 (1.20–2.02)	1.70 (1.20–2.50)	2.00 (1.15–3.10)	**0.003**
**Vitamin D [ng/mL]**	116	22 (15–24)	17 (13–21)	21 (13–36)	19 (14–29)	21 (14–24)	0.5
**Lactic acid [mmol/L]**	86	3.20	1.35 (1.10–1.70)	1.30 (1.10–1.80)	1.60 (1.20–2.40)	1.65 (1.30–2.10)	0.2
**Uric acid [μmol/L]**	125	277 (209–372)	290 (227–342)	307 (210–382)	326 (149–401)	282 (163–373)	>0.9
**Thyroid-stimulating hormone**	100	1.35 (1.18–1.96)	2.26 (0.99–2.79)	1.94 (1.57–2.38)	1.69 (0.45–2.23)	1.40 (0.43–3.51)	0.5
**Free Thyroxine**	96	13.0 (11.3–14.7)	14.1 (11.6–15.2)	14.7 (12.5–17.3)	16.6 (14.4–19.1)	12.8 (9.1–15.1)	**0.002**
**Vitamin B12**	66	232 (120–344)	239 (192–361)	242 (174–447)	455 (284–612)	627 (350–815)	**0.003**

^1^ Median (Q1–Q3); n (%). ^2^ Kruskal–Wallis rank sum test; Fisher’s exact test. Values are presented as median (interquartile range, Q1–Q3) for continuous variables and number (percentage) for categorical variables. Disease severity is as per Table 1. “NA” indicates data not available for that group. *p*-values represent overall comparisons across severity groups using the Kruskal–Wallis rank sum test for continuous variables and Fisher’s exact test for categorical variables.

**Table 3:** ACE2 levels in patients with COVID-19. Circulating ACE2 levels in patients with COVID-19 stratified by disease severity.

Group	N	Minimum (pg/mL)	Maximum (pg/mL)	Mean (pg/mL)	Std. Deviation	Median (pg/mL)	Adjusted *p*-Value
**Asymptomatic**	38	0.00	1407.67	145.66	259.16	54.57	-
**Mild symptomatic**	41	0.00	435.51	81.60	92.74	57.15	1.000 *
**Mild pneumonia**	41	0.00	123.17	24.93	25.76	18.11	<0.001 *
**Severe Illness**	48	0.00	691.83	46.73	109.33	16.22	<0.001 *
**Critical Illness**	56	0.00	337.29	16.67	48.24	0.00	<0.001 *
**Total**	224	0.00	1407.67	58.39	134.04	20.05	

The table summarizes plasma ACE2 concentrations (pg/mL) in COVID-19 patients categorized by clinical severity (asymptomatic, mild symptomatic, mild pneumonia, severe illness, and critical illness). Data are presented as minimum, maximum, mean, standard deviation, and median values for each group, along with sample size (N). Adjusted *p*-values indicate between-group comparisons relative to the asymptomatic group, calculated using Dunn’s post hoc test with Bonferroni correction following non-parametric analysis. Statistically significant differences compared with the asymptomatic group are indicated (*).

**Table 4:** Levels of angiotensin-converting enzyme (ACE) in patients with COVID-19. Circulating ACE levels in patients with COVID-19 by disease severity.

Group	N	Minimum (ng/mL)	Maximum (ng/mL)	Mean (ng/mL)	Std. Deviation	Median (ng/mL)	*p*-Value
**Asymptomatic**	38	19.83	137.99	70.71	29.43	64.52	-
**Mild Symptomatic**	41	15.07	173.01	62.88	35.11	53.42	1.000 *
**Mild pneumonia**	41	23.68	169.72	88.70	34.96	85.49	0.603 *
**Severe Illness**	48	38.67	194.24	116.82	40.56	116.18	<0.001 *
**Critical Illness**	56	81.64	261.61	143.69	37.19	139.23	<0.001 *
**Total**	224	15.07	261.61	100.70	47.27	95.52	

Plasma ACE concentrations (ng/mL) are shown across clinical severity groups as minimum, maximum, mean, standard deviation, and median values with corresponding sample sizes (N). *p*-values represent comparisons with the asymptomatic group using Dunn’s test with Bonferroni correction; * denotes statistical significance.

**Table 5:** ACE/ACE2 ratio in patients with COVID-19. ACE/ACE2 ratio in patients with COVID-19 across disease severity.

Group	N	Minimum	Maximum	Mean	Std. Deviation	Median	Adjusted *p*-Value
**Asymptomatic**	38	0.04	105.38	4.33	16.90	1.16	-
**Mild symptoms**	41	0.07	119.42	10.68	28.80	0.87	1.000 *
**Mild pneumonia**	41	0.38	136.30	22.65	39.17	4.57	0.001 *
**Severe illness**	48	0.13	192.92	26.97	49.38	6.95	<0.001 *
**Critical illness**	56	0.47	261.61	93.64	76.58	110.17	<0.001 *
**Total**	224	0.04	261.61	36.02	60.04	4.46	

The ACE/ACE2 ratio is summarized by clinical severity group using descriptive statistics (minimum, maximum, mean, standard deviation, and median) and sample size (N). Adjusted *p*-values indicate comparisons with the asymptomatic group using Dunn’s test with Bonferroni correction; * denotes statistical significance.

## Data Availability

The data that support the findings of this study are available on request from the corresponding author.

## Supplementary Materials

The following supporting information can be downloaded at: https://www.mdpi.com/article/10.3390/v18040465/s1, Table S1: Prevalence of key comorbidities stratified by COVID-19 disease severity.
