# Supplementary material for: Plasma ACE and ACE2 Levels Are Altered in Patients with COVID-19
Source: Viruses. 2026 Apr 14;18(4):465. doi: 10.3390/v18040465 (PMC13119688; doi:10.3390/v18040465)
Supplement: Supplementary file 1 [file viruses-18-00465-s001.zip › viruses-4221553-supplementary.pdf]

Table S1. Prevalence of key comorbidities stratified by COVID-19 disease severity

Severity	N	HTN n (%)	Diabetes mellitus n (%)	Cardiovascular disease n (%)	Chronic kidney disease n (%)	Chronic liver disease n (%)
**Asymptomatic**	38	13 (34.2)	13 (34.2)	0 (0.0)	0 (0.0)	0 (0.0)
**Mild Symptomatic**	41	11 (26.8)	13 (31.7)	3 (7.3)	0 (0.0)	0 (0.0)
**Mild Pneumonia**	41	18 (43.9)	21 (51.2)	1 (2.4)	2 (4.9)	0 (0.0)
**Severe**	48	28 (58.3)	23 (47.9)	6 (12.5)	4 (8.3)	2 (4.2)
**Critical**	56	39 (69.6)	36 (64.3)	18 (32.1)	14 (25.0)	6 (10.7)
**p-value**		<0.001	0.007	<0.001	<0.001	0.012

P-values are from Fisher's exact tests comparing prevalence across severity groups.

Comorbidities were markedly more prevalent in the mild-pneumonia, severe, and critical groups, consistent with known risk factors for COVID-19 progression.
